# Salbutamol-responsive limb-girdle congenital myasthenic syndrome due to a novel missense mutation and heteroallelic deletion in MUSK

**DOI:** 10.1016/j.nmd.2013.08.002

**Published:** 2014-01

**Authors:** Constanze Gallenmüller, Wolfgang Müller-Felber, Marina Dusl, Rolf Stucka, Velina Guergueltcheva, Astrid Blaschek, Maja von der Hagen, Angela Huebner, Juliane S. Müller, Hanns Lochmüller, Angela Abicht

**Affiliations:** a Friedrich-Baur-Institut, Ludwig Maximilians University, Munich, Germany; b Haunersche Kinderklinik, Ludwig Maximilians University, Munich, Germany; c Clinic of Neurology, University Hospital Alexandrovska, Sofia, Bulgaria; d Children’s Hospital, Technical University Dresden, Germany; e Institute of Genetic Medicine, Newcastle University, Newcastle upon Tyne, United Kingdom; f Medical Genetics Center, Munich, Germany

**Keywords:** AChR, acetylcholine receptor, AChE, acetylcholine esterase, CK, creatine kinase, CMAP, compound muscle action potential, CMS, congenital myasthenic syndrome, Congenital myasthenic syndromes, Neuromuscular transmission, MUSK-related CMS, Salbutamol treatment

## Abstract

Congenital myasthenic syndromes (CMS) are clinically and genetically heterogeneous disorders characterized by a neuromuscular transmission defect. In recent years, causative mutations have been identified in atleast 15 genes encoding proteins of the neuromuscular junction. Mutations in *MUSK* are known as a very rare genetic cause of CMS and have been described in only three families, world-wide. Consequently, the knowledge about efficient drug therapy is very limited. We identified a novel missense mutation (p.Asp38Glu) heteroallelic to a genomic deletion affecting exons 2–3 of *MUSK* as cause of a limb-girdle CMS in two brothers of Turkish origin. Clinical symptoms included fatigable limb weakness from early childhood on. Upon diagnosis of a *MUSK*-related CMS at the age of 16 and 13 years, respectively, treatment with salbutamol was initiated leading to an impressive improvement of clinical symptoms, while treatment with esterase inhibitors did not show any benefit. Our findings highlight the importance of a molecular diagnosis in CMS and demonstrate considerable similarities between patients with MUSK and DOK7-related CMS in terms of clinical phenotype and treatment options.

## 1 Introduction

Congenital myasthenic syndromes (CMS) comprise a group of hereditary disorders characterized by a neuromuscular transmission defect. In recent years, disease-associated mutations have been identified in several genes that encode different presynaptic, synaptic, and postsynaptic proteins of the neuromuscular junction [1–3]. Postsynaptic CMS caused by mutations identified in *RAPSN*, *DOK7*, *AGRN*, and *MUSK* further underlined the key role of the agrin–MuSK–rapsyn pathway for the organization and maintenance of the postsynaptic scaffold [4–6]. MuSK, the muscle-specific receptor tyrosine kinase, plays a central signaling role in this pathway and is essential for prepatterning acetylcholine receptors (AChRs) in muscle prior to innervation and for agrin-induced AChR clustering: Agrin, a heparin sulfate proteoglycan supplied by motor neurons, binds to LRP4 (low density lipoprotein receptor related protein 4) expressed in muscle [7–9], leading to activation of MuSK [10]. In the developing nerve terminal, activation of MuSK is a critical step and results in the recruitment of several downstream kinases, clustering of essential postsynaptic proteins including Rapsyn and the AChRs, synapse-specific gene transcription and the generation of retrograde signals that regulate presynaptic differentiation [11–13].

The MuSK protein includes a large ectodomain containing IgG-like motif, a transmembrane helix and a cytoplasmic tyrosine kinase domain. Its activation is characterized by an induced autophosphorylation and an increased kinase activity. MuSK requires a dual activation, extracellularly by agrin and intracellularly by Dok7. Agrin does not interact directly with MuSK but binds LRP4. LRP4 and MuSK interact via their respective extracellular domains [9,14]. The crystal structure of MuSK and Dok7 revealed that the phosphotyrosine-binding domain (PTB domain) of Dok7 forms an integrated, dimeric structural unit that dimerizes MuSK and thereby facilitates trans-autophosphorylation of tyrosines in the MuSK activation loop [15].

The gene *MUSK* is located on chromosome 9q31.3-q32 and comprises 14 exons. Autosomal recessive *MUSK* mutations have been first identified in a single CMS patient [16] harbouring two heteroallelic mutations, a frameshift mutation (c.220insC) and a missense mutation (p.V790M). We recently reported on CMS caused by a homozygous missense mutation (p.P344R) in *MUSK* in five affected sibs from a consanguineous Sudanese family. The affected proline is located in the Frizzled-like cysteine-rich domain, a part of the extracellular domain of MuSK [17]. In addition, two missense mutations (p.M605I and p.A727V), both located in the kinase domain of MuSK have been identified, recently [18].

## 2 Materials and methods

### 2.1 Patients, DNA samples, linkage analysis, sequencing

We analyzed two affected brothers and their unaffected parents by linkage analysis and direct sequencing as previously described [19]. After reducing the number of candidate genes using a linkage approach with DNA marker sets of the known CMS and congenital myopathy genes [20] several known genes to cause CMS when mutated (*COLQ*, *DOK7, RAPSN, CHAT, CHRNE, CHRNA1, CHRND, CHRNE, GFPT1, and MUSK*) were analyzed in the younger brother as in the index patient. Paternity was confirmed by a set of highly polymorphic microsatellite markers using the “Identifiler-plus”-Kit (Applied Biosystems, Carlsbad, California).

### 2.2 cDNA analysis

A muscle biopsy specimen was obtained from the elder brother. RNA was isolated from muscle tissue using the RNeasy Mini Kit (QIAGEN). cDNA was synthesized from RNA using the Transcriptor One-Step RT-PCR Kit with exon-specific primers of the *MUSK* gene according to the manufacturer’s recommendations (exon 1: 5′-CCTTCAGCGGAACTGAGAAA-3′ and exon 4: 5′-TGGTTTGGGATTACCCATTG-3′). Corresponding PCR products were analyzed by agarose gel electrophoresis, excised from the gel, and analyzed by direct sequencing.

## 3 Results

### 3.1 Case report

**Fig. 1:**
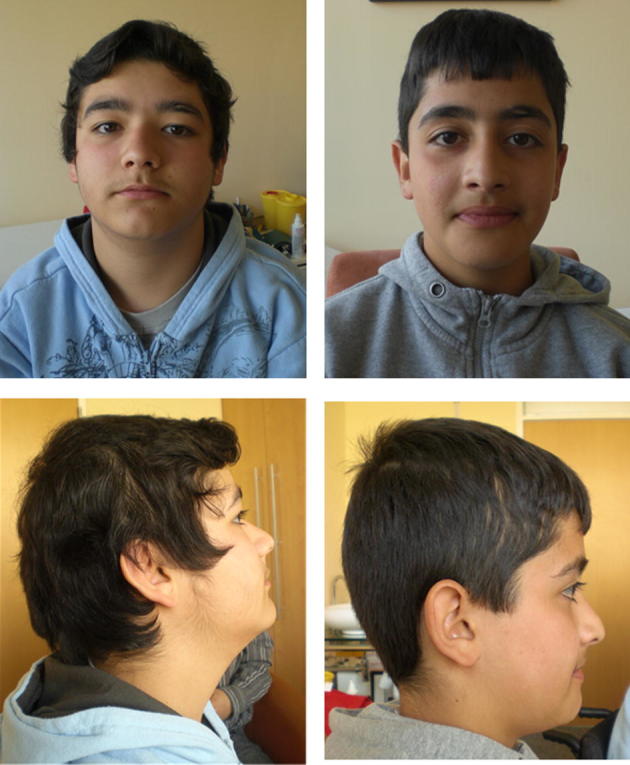
The two brothers before salbutamol treatment at age 15 and 12 years, respectively. No obvious ptosis is present in both siblings.

We report on two affected brothers from a Turkish non consanguineous family. After having achieved motor milestones at time and walking independently at the age of 12 months, his mother noted that her first child (patient 1) used furniture to pull himself up to a stand. Over the next few years a reduced exercise tolerance became more obvious. When the older brother presented to the neuropediatric department at the age of 3.5 years, an abnormal exercise tolerance was also noted in the younger brother (patient 2) who was nine months old at this time. Subsequent follow-up revealed a progressive worsening of muscle endurance in both patients leading to an inability to walk in the afternoon in patient 1 at the age of 15 years. The younger brother is ambulatory and able to climb stairs at school but is easily exhausted. Both brothers manifest a proximal limb and facial muscle weakness without any bulbar and ocular symptoms such as ptosis or limitation of eye movements (Fig. 1). A muscle biopsy at age 9 years in the elder brother revealed minimal myopathic changes. Sonographic examination of the muscle showed an increased echo signal of the quadriceps femoris muscle in patient 1 and a normal pattern in his brother.

At the age of 11 years, repetitive stimulation of the accessorius nerve in patient 1 resulted in a 19% decrement of the CMAP. Repetitive CMAPs were not observed after a single nerve stimulus. CK levels were normal, and serum antibodies against AChR negative. A trial with oral pyridostigmine bromide in patient 1 at the age of 10 years resulted in a worsening of the symptoms and was stopped after 3 weeks of treatment. A second trial with pyridostigmine showed a similar effect. Salbutamol was tried at the age of 16 and 13 years, respectively, in both patients. At that time patient 1 was unable to get out of the wheelchair without support in the afternoon. Patient 2 had to use a bannister to climb stairs in the afternoon. One week after starting salbutamol (2 × 4 mg/day) patient 1 was able to climb stairs again using a bannister. Patient 2 was able to climb stairs without any aids. There was a further improvement of symptoms after increasing the daily dosage of salbutamol to 3 × 4 mg in both brothers.

### 3.2 Mutation analysis

**Fig. 2:**
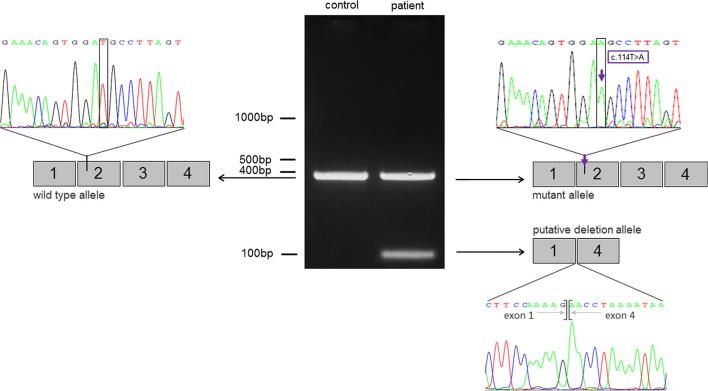
Detection of the putative deletion allele by PCR amplification of cDNA derived from muscle tissue RNA of the older brother (patient 1). The expected product of 400 bp in length was found in the control sample and in the patient. The heteroallelic deletion detected in the patient is represented by an additional PCR product of 100 bp. The sequence analysis of the 100 bp-band revealed a lack of exons 2–3 and exon 1 is spliced to exon 4. Sequence analysis revealed that the mutation c.114T > A was only present in the longer PCR-product of the patient but not in the short product. The presence of putative intronic splice-site mutations was excluded by sequencing 100 bp of the 5′-prime and 3′-prime flanking regions of exons 1–4.

Sequence analysis of the coding region of *MUSK* revealed a seemingly homozygous T to A transition in exon 2 at nucleotide position 114 leading to an amino acid exchange (p.Asp38Glu) in both affected brothers. The sequence variant was not identified in the relevant SNP databases including 5000 exomes, and not in 50 ethnically matched control chromosomes. Segregation analysis in the family revealed that the mutation was present heterozygously in the mother but was not present in the father. We hypothesized that a heteroallelic deletion may have been missed by our analysis of genomic DNA. PCR amplification of cDNA derived from muscle tissue RNA of the older brother by a set of primers spanning exon 1 to exon 4 of *MUSK* revealed the presence of an additional band of 100 bp which was shorter than the expected 400 bp wild-type product. In the sequencing reaction of the 100 bp-band exons 2–3 were missing and exon 1 was spliced to exon 4 (Fig. 2).

## 4 Discussion

Mutations in *MUSK* are known as a very rare genetic cause of CMS and have been described in only three families, so far. We identified a novel missense mutation c.114T > A; p.Asp38Glu heteroallelic to a genomic deletion encompassing exons 2–3 of *MUSK* detected by polymerase chain reaction (PCR)-based methods that explain a limb-girdle CMS in two affected brothers of a Turkish family.

The patients’ fatigable and varying weakness presenting as a limb-girdle phenotype and the decremental response of the CMAP on repetitive stimulation were consistent with a myasthenic phenotype. However, the absence of ocular symptoms, the ineffectiveness of pyridostigmine treatment, and the absence of genetic findings made the distinction to a congenital myopathy challenging.

The clinical phenotype in our patients largely resembles patients with DOK7 mutations including the pronounced weakness of limb girdle musculature, normal eye movements, and ineffectiveness of esterase inhibitors. The clinical characteristics together with the mutations in the *MUSK* gene suggested a therapeutic trial with salbutamol that led to a dramatic clinical improvement of the patientś symptoms within a week after years of moderate disease progression. The clinical phenotype we have seen in our patients has been described in the few previously described *MUSK* patients. We may hypothesize that MuSK deficiency, similar to Dok7 deficiency, results in a defect of synapse formation or maintenance (“synaptopathy”) which does not respond to esterase inhibitor treatment. While mutations in *MUSK* are found less frequently than *DOK7* mutations, we would suggest testing of the *DOK7* gene first, and then the *MUSK* gene, in patients with limb-girdle CMS unresponsive to esterase inhibitors. In contrast, most patients with LG-CMS and tubular aggregates responding to esterase inhibitors carry mutations in *GFPT1* or *DPAGT*.

In CMS patients due to *COLQ*, *DOK7* and *LAMB2* mutations sympathomimetics like ephedrine and salbutamol have been shown to have a clear beneficial effect without major side effects [21,22]. In contrast, only moderate improvement with albuterol sulfate and no improvement with ephedrine has previously been reported for a single patient with CMS caused by two missense mutations in *MUSK* [18]. A dramatic clinical effect of salbutamol treatment is a novel finding for patients with *MUSK*-related CMS. However, the mode of action of sympathomimetic agents in some forms of CMS is unknown.

The mutation c.114T > A; p.Asp38Glu identified in our patients is not described as a pathogenic mutation or as a polymorphism. Pathogenicity is supported by the segregation of the mutation with the disease phenotype in the family and by its absence from 50 ethnically matched control chromosomes and relevant databases including 5000 exomes. A bioinformatic analysis using PolyPhen-2 (http://genetics.bwh.harvard.edu/ggi/pph2) [23] predicts the exchange of the highly conserved amino acid Asp to be probably damaging with a score of 0.988 (sensitivity: 0.72; specificity: 0.96). Three of the four previously reported missense mutations are located in the C-terminal domain of the protein corresponding to the conserved catalytic tyrosine kinase domain: V790M [16], M605I and A727V [18]. Further functional studies for those mutations indicated the inability of MuSK mutants to interact with Dok-7 as a major determinant of the molecular pathogenesis [16,18,24]. In contrast to three of the four previously reported missense mutations, our missense mutation p.Asp38Glu reported is located in the extracellular protein domain, even closer to the N-terminus than the missense mutation p.P344R previously reported by us [17]. Functional studies have not been carried out for either of the N-terminus mutations. It can be speculated that mutations of the extracellular part of the protein do not affect the interaction with Dok-7 which takes part intracellularly [15] but the extracellular activation effected by agrin. The affected aspartate at position 38 is part of a beta-strand of Ig-like domain 1 (Ig1) of the MuSK extracellular region that is known to form a homodimer mediated by a hydrophobic face [25]. Thus, the missense mutation p.Asp38Glu might interfere with the dimerization of MuSK and the subsequent association with LRP4 to form a functional LRP4-MuSK complex [8,9]. Recent studies suggest a role of Wnt in synapse formation. Interestingly, there is also indication that the extracellular domain of MuSK is involved in Wnt binding [26,27]. Alternatively, the mutation might cause misfolding of Ig1, leading to retention of MuSK inside the cell. However, as functional studies have not been carried out this remains speculative. The second mutation identified in our patients is a two-exons deletion. In general, genomic deletions are relatively common, but often not detected by standard diagnostic sequencing. Our finding of a genomic deletion of MUSK further emphasizes the importance of dosage sensitive screening techniques in molecular genetic testing.
